# Supplementary material for: Scaling of an antibody validation procedure enables quantification of antibody performance in major research applications
Source: bioRxiv. 2023 Aug 17:2023.06.01.543292. Preprint. [Version 6] doi: 10.1101/2023.06.01.543292 (PMC10312534; doi:10.1101/2023.06.01.543292)
Supplement: Supplement 3 [file NIHPP2023.06.01.543292v6-supplement-3.pdf]

bioRxiv preprint doi: https://doi.org/10.1101/2023.06.01.543292; this version posted August 17, 2023. The copyright holder for this preprint (which was not certified by peer review) is the author/funder, who has granted bioRxiv a license to display the preprint in perpetuity. It is made available under aCC-BY 4.0 International license.

**Supplemental Figure 1: Schematic representations of antibody performance**

(**A**) Schematic representations of a successful antibody (left schematic), specific, non-selective antibody (middle schematic), and a non-successful antibody (right schematic) for WB. (**B**) Schematic representations of a successful antibody (left schematic) and non-successful antibodies (middle and right schematics) for IP. (**C**) Schematic representation of the mosaic strategy used (left schematic). WT cells are labelled with a fluorescent cell dye (green), and KO cells are labelled with a different fluorescent cell dye (magenta) plated together as a mosaic. Schematic representations of a successful antibody (antibody #1) and a non-successful antibody (antibody #2) for IF are shown.

bioRxiv preprint doi: https://doi.org/10.1101/2023.06.01.543292; this version posted August 17, 2023. The copyright holder for this preprint (which was not certified by peer review) is the author/funder, who has granted bioRxiv a license to display the preprint in perpetuity. It is made available under aCC-BY 4.0 International license.

**Supplemental Figure 2: Analysis of antibody performance by manufacturer's catalogue recommendation**

Percentage of successful or unsuccessful antibodies for the indicated applications are shown using a bar graph with stacked columns. Antibodies were divided according to whether they were recommended or not recommended by the manufacturers for the indicated applications. The percentage corresponding to each section of the bar graph is shown in the graph, and the total number of antibodies represented in each category is indicated above the corresponding bar.

**Supplemental Figure 3: Actions taken from participating companies**

The percentage of antibodies removed from the market, or for which catalogue recommendations were modified following assessment of our data by our antibody manufacturing partners. The number of antibodies represented in each category is indicated above the corresponding bar.

**Supplemental Figure 4: Correlation of antibody performance between applications**

(**A**) Representation of a 2 x 2 contingency table used to apply the McNemar Test as well as the equation of the chi-square ($X^2$) statistic used. Analysis of antibody performance correlation, represented as a contingency table and as a double y-axis graph between (**B**) WB and IP, (**C**) IF and IP and (**D**) IF and WB. n/s = non-significant

bioRxiv preprint doi: https://doi.org/10.1101/2023.06.01.543292; this version posted August 17, 2023. The copyright holder for this preprint (which was not certified by peer review) is the author/funder, who has granted bioRxiv a license to display the preprint in perpetuity. It is made available under aCC-BY 4.0 International license.

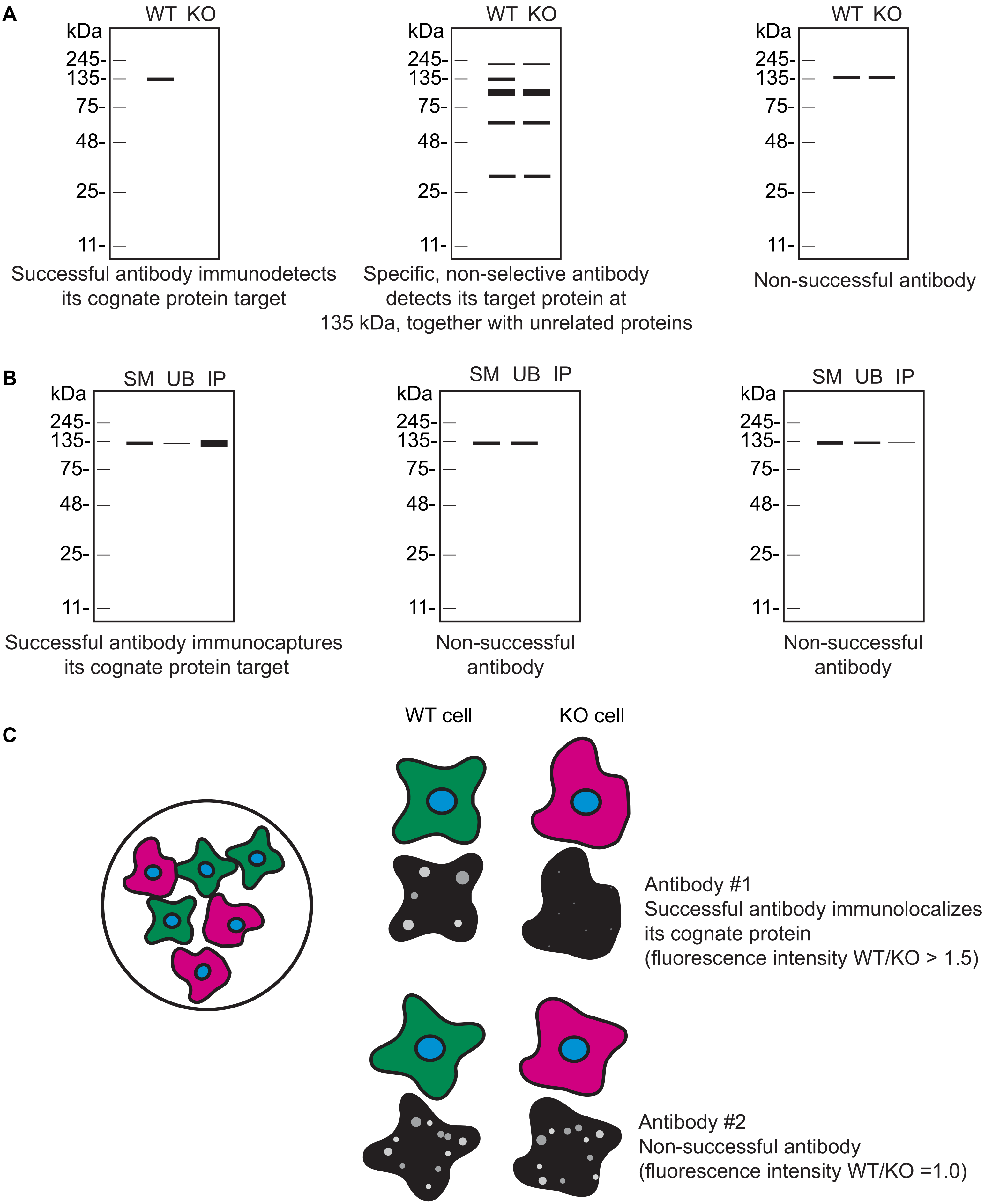


**Ayoubi et al., Supplemental Figure 1**

bioRxiv preprint doi: https://doi.org/10.1101/2023.06.01.543292; this version posted August 17, 2023. The copyright holder for this preprint (which was not certified by peer review) is the author/funder, who has granted bioRxiv a license to display the preprint in perpetuity. It is made available under aCC-BY 4.0 International license.

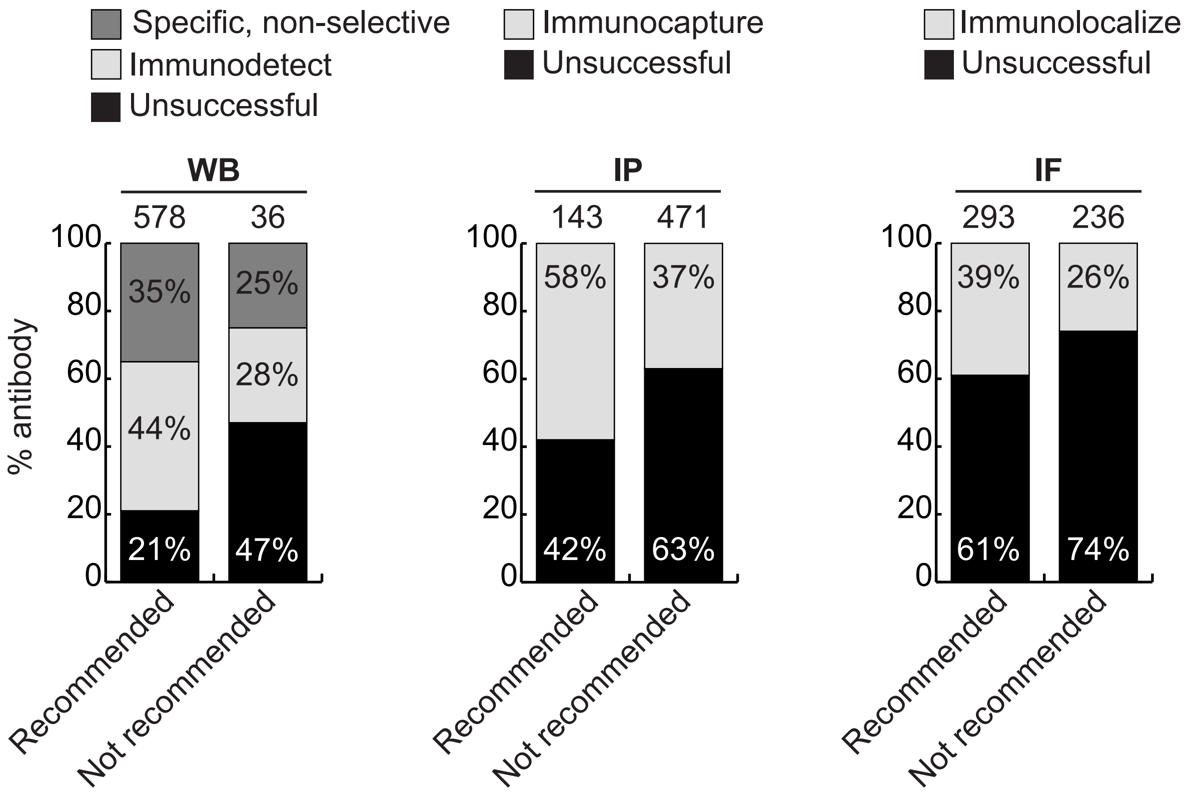


**Ayoubi et al., Supplemental Figure 2**

bioRxiv preprint doi: https://doi.org/10.1101/2023.06.01.543292; this version posted August 17, 2023. The copyright holder for this preprint (which was not certified by peer review) is the author/funder, who has granted bioRxiv a license to display the preprint in perpetuity. It is made available under aCC-BY 4.0 International license.

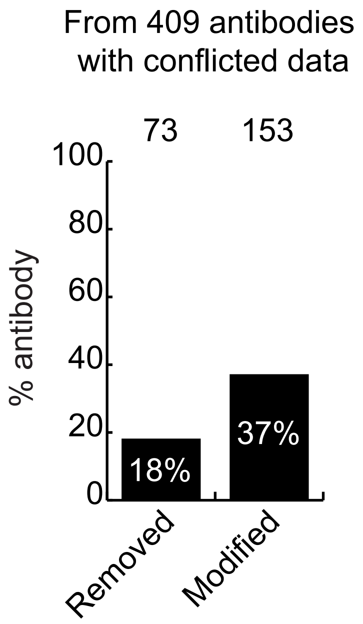


**Ayoubi et al., Supplemental Figure 3**

bioRxiv preprint doi: https://doi.org/10.1101/2023.06.01.543292; this version posted August 17, 2023. The copyright holder for this preprint (which was not certified by peer review) is the author/funder, who has granted bioRxiv a license to display the preprint in perpetuity. It is made available under aCC-BY 4.0 International license.

**A**

application #2 (columns); application #1 (rows)

| | fail | pass |
|---|---|---|
| fail | a | b |
| pass | c | d |

$$\chi^2 = \frac{(b-c)^2}{b+c}$$

**B**

| | IP: fail | IP: pass |
|---|---|---|
| WB: fail | 190 | 101 |
| WB: pass | 126 | 112 |

$\chi^2=1.3$
$p>0.1$

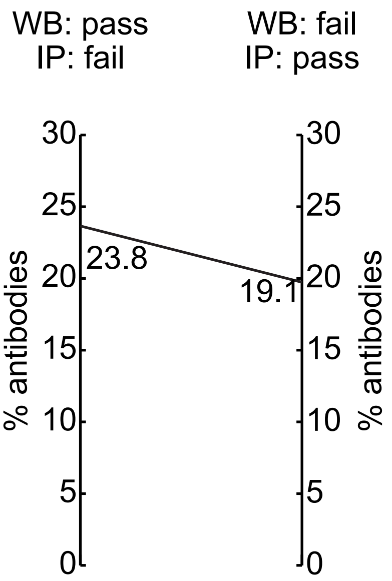


**C**

| | IP: fail | IP: pass |
|---|---|---|
| IF: fail | 255 | 98 |
| IF: pass | 61 | 115 |

$\chi^2=8.6$
$p<0.005$

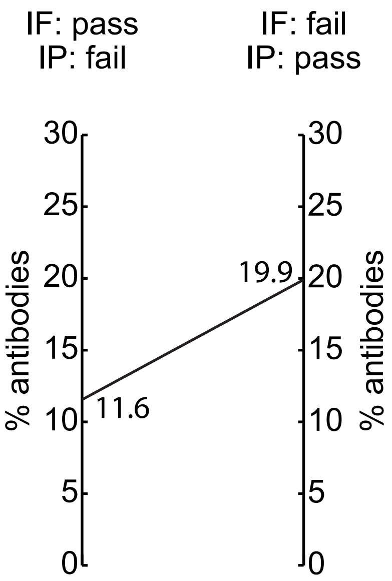


**D**

| | WB: fail | WB: pass |
|---|---|---|
| IF: fail | 232 | 121 |
| IF: pass | 59 | 117 |

$\chi^2=21.4$
$p<0.0005$

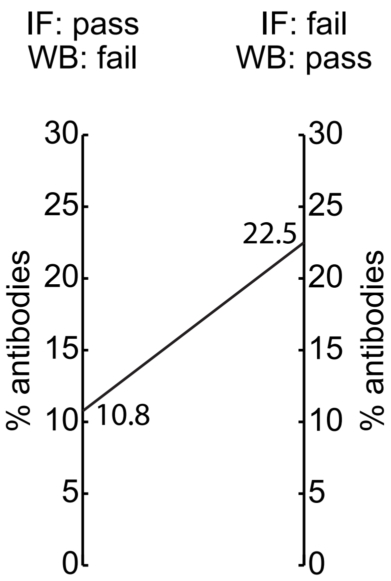


**Ayoubi et al., Supplemental Figure 4**
